# General Method for the Synthesis of (−)-Conduritol C and Analogs from Chiral Cyclohexadienediol Scaffolds

**DOI:** 10.3390/molecules23071653

**Published:** 2018-07-06

**Authors:** Gaurao D. Tibhe, Mario A. Macías, Valeria Schapiro, Leopoldo Suescun, Enrique Pandolfi

**Affiliations:** 1 Laboratorio de Síntesis Orgánica, Departamento de Química Orgánica, Facultad de Química, Av. Gral. Flores 2124, Universidad de la República, Montevideo CP11800, Uruguay; gaurao.tibhe@gmail.com (G.D.T.); vschapir@fq.edu.uy (V.S.); 2 Cryssmat-Lab/DETEMA, Facultad de Química, Av. Gral. Flores 2124, Universidad de la República, Montevideo CP11800, Uruguay; ma.maciasl@uniandes.edu.co (M.A.M.); Leopoldo@fq.edu.uy (L.S.); 3 Department of Chemistry, Universidad de los Andes, Carrera 1 N8 18 A-12, Bogotá 111711, Colombia

**Keywords:** conduritol C, enantioselective biocatalysis, arene biotransformation, crystal structures, α-glycosidase inhibitors

## Abstract

An efficient and facile general method for the synthesis of conduritol C analogs, taking advantage of an enantioselective biocatalysis process of monosubstituted benzenes, is described. The absolute stereochemical patterns of the target molecules (−)-conduritol C, (−)-bromo-conduritol C, and (−)-methyl-conduritol C were achieved by means of chemoenzymatic methods. The stereochemistry present at the homochiral cyclohexadiene-*cis*-1,2-diols derived from the arene biotransformation and the enantioselective ring opening of a non-isolated vinylepoxide derivative permitted the absolute configuration of the carbon bearing the hydroxyl groups at the target molecules to be established. All three conduritols and two intermediates were crystallized, and their structures were confirmed by X-ray diffraction. The three conduritols and intermediates were isostructural. The versatility of our methodology is noteworthy to expand the preparation of conduritol C analogs starting from toluene dioxygenase (TDO) monosubstituted arene substrates.

## 1. Introduction

The synthesis of conduritol C, one of the cyclohex-5-ene-1,2,3,4-tetrol isomers named conduritols by Kübler in 1908 [1], has been extensively reported, although only conduritol A and F occur in nature. Since the first racemic synthesis of conduritol C from bromo *epi*-inositol by McCasland and Reeves [2], several groups have proposed isolated efforts toward this goal [3,4,5]. A chemoenzymatic strategy to obtain enantiomerically pure conduritol C from cyclohexadiene-*cis*-1,2-diols has also been reported [6,7,8,9,10]. In addition, halo-conduritols have attracted researchers’ attention [8,11], and recently they have been found to possess enzyme-specific inhibition against α-glycosidase [11]. To the best of our knowledge, there is no report of the synthesis or structural characterization of methyl-conduritol C. 

**Scheme 1:**
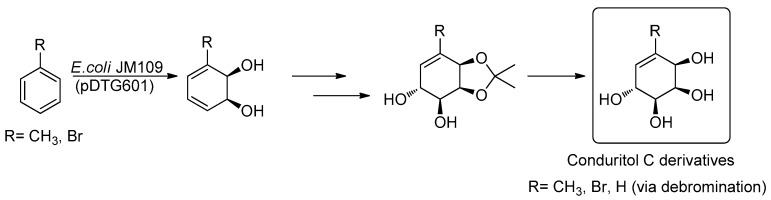
Synthetic strategy for the preparation of conduritol C derivatives.

The present work reports a general method for the synthesis of enantiopure conduritol C derivatives. We describe the enantiocontrolled process from homochiral substituted *cis*-1,2-cyclohexadienediols obtained by biotransformation to the target derivatives in four steps (see Scheme 1). 

Additionally, the crystal structures of methyl-conduritol C, bromo-conduritol C, and H conduritol C, as well as acetonide derivatives of the last two compounds, are reported, complementing the already reported methyl-conduritol acetonide structure [12]. Both groups of three compounds showed isostructural crystals that suggest that modification of the substituent at C5/C7 in the conduritols/acetonides (CH_3_, Br, or H) is not involved in intermolecular interactions that determine the crystal packing. 

## 2. Results and Discussion

### 2.1. Synthetic Strategy

The synthetic strategy for the construction of conduritol C stereochemical patterns takes advantage of the enzymatic *cis*-dihydroxylation process from substituted arenes that generates two of the required stereocenters. This method, previously reported by our lab, produces the corresponding *cis*-diol by microbial oxidation using the mutant bacteria *E. coli* JM109 (pDTG601) with excellent enantiomeric excess (>99% ee) (see Scheme 1) [13,14].

**Scheme 2:**
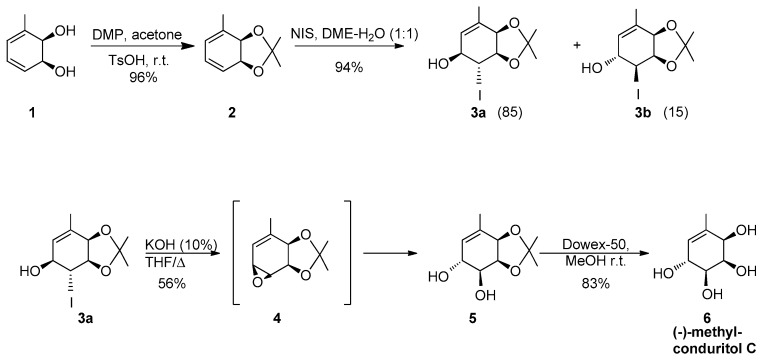
Synthesis of methyl-conduritol C.

**Figure 1:**
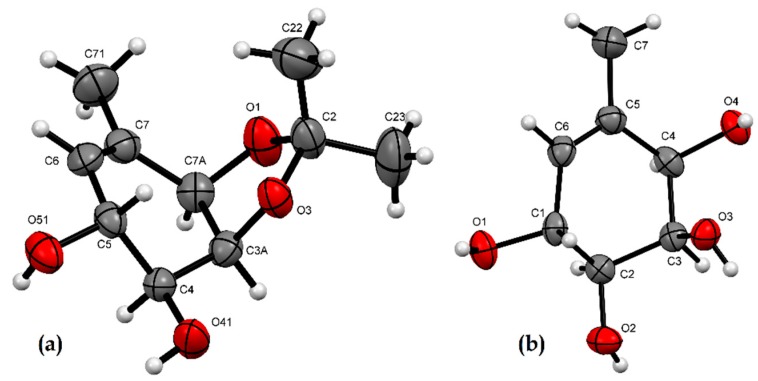
Oak Ridge Thermal Ellipsoid Plot (ORTEP) plots of the crystal structures of (**a**) methyl-conduritol C acetonide (**5**) [12], and (**b**) (−)-methyl-conduritol C (**6**).

Starting from methyl-cyclohexadienediol **1** (23 g/L from toluene) [14] (Scheme 2), we recently reported the synthesis [15] of **5** in four steps with an overall 24% yield, and we also described its crystal structure (Figure 1a) [12]. We optimized the reported method as follows: The reaction of acetonide **2** under Prevost conditions [16,17] produced iodohydrin **3a** as the major product, which, treated with an excess of aqueous KOH at reflux, generated diol **5**, without isolation of epoxide **4**. In the previous report, we isolated epoxide **4,** and we then treated it with 10% KOH in tetrahydrofurane (THF) under reflux, providing diol **5**. By utilizing the present method, we achieved a 56% yield in a one-pot reaction, which represents a more than 2-fold increased yield compared with the previous strategy. Finally, removal of the acetonide on compound **5** by using Dowex 50 overnight at room temperature afforded methyl-conduritol C in an 83% yield (Scheme 2, Figures S1 and S2). The structure of **6** was also confirmed by single-crystal X-ray diffraction analysis (Figure 1b). 

**Scheme 3:**
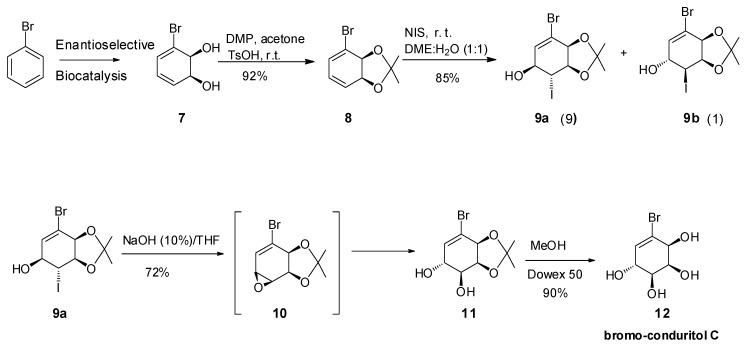
Synthesis of bromo-conduritol C.

Our general method leads to secured stereochemistry at all four stereogenic centers in the conduritol C derivatives. Bromo-cyclohexadienediol **7** (43 g/L) [14] (Scheme 3), which was obtained by biotransformation of bromobenzene, established the absolute configuration of two stereogenic centers that we protected under the conventional protocol to the corresponding dioxolane **8**.

**Figure 2:**
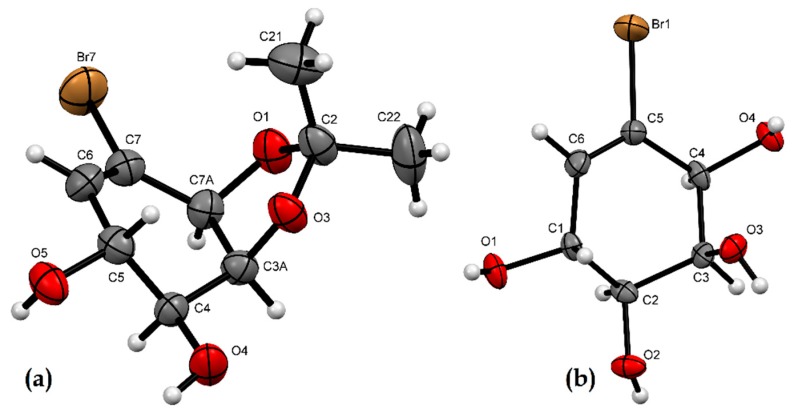
ORTEP plots of (**a**) bromo-conduritol C acetonide (**11**), and (**b**) (−)-bromo-conduritol C (**12**).

Similarly, the treatment of **8** under Prevost conditions resulted in the formation of isomer **9a** (major product), as reported earlier [17]. Complete stereocontrol in the introduction of the following hydroxyl groups could be achieved by a direct attack with 10% aqueous NaOH in THF to the vinylic epoxide intermediate **10**, leading to enantiomerically pure compound **11** [18]. Its absolute stereochemistry was confirmed by X-ray diffraction analysis (Figure 2a). Compound **11**, having the right stereochemistry in all its stereogenic centers, was deprotected under conventional methods using Dowex 50 in methanol. Bromo-conduritol C (90%) was thus obtained without epimerization (see Figures S3 and S4), as confirmed by X-ray diffraction analysis of bromo-conduritol C **12** (see Figure 2b).

**Scheme 4:**
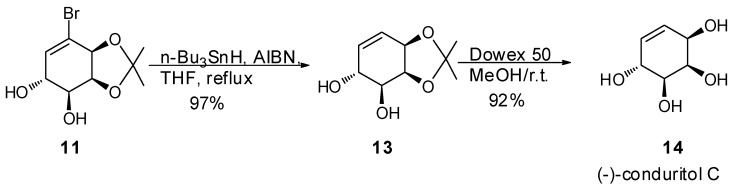
Synthesis of (−)-conduritol C.

**Figure 3:**
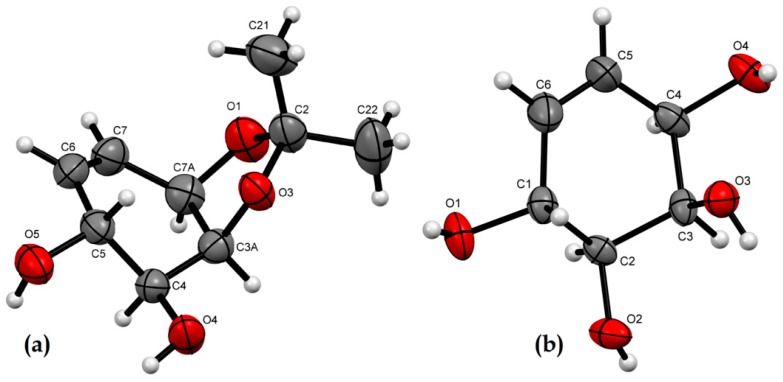
ORTEP plots of (**a**) conduritol C acetonide (**13**), and (**b**) (−)-conduritol C (**14**).

According to Hudlicky et al. [19], the bromo-conduritol precursor **11** was reductively dehalogenated under free-radical conditions (see Scheme 4) to afford compound **13**, which was deprotected to afford (−)-conduritol C. Thus, we obtained (−)-conduritol C along the same synthetic avenue in five steps with a 45% yield. The crystal structures of **13** and **14** were also determined, as shown in Figure 3a,b, respectively. 

### 2.2. X-ray Crystallographic Analysis

The crystal structures of acetonides **11** and **13** are discussed first as a group, as they showed identical molecular conformations as well as identical intermolecular interactions. After this, the structures of **6**, **12**, and **14** are discussed, also taking advantage of their isostructurality.

Compound **13** crystallized in the orthorhombic P2_1_2_1_2_1_ space group with a = 6.1358(5) Å, b = 7.5083(5) Å, c = 20.5776(14) Å, and V = 948.00(12), similarly to compound **5** [12], while **11** crystallized in the monoclinic P2_1_ space group with a = 6.1570(2), b = 7.4749(3), c = 11.7514(4), β = 93.267(3)°, and V = 539.96(3) (see Tables S1 and S2 for detailed crystallographic information). Comparing the three molecules of **5**, **11**, and **13** (see Figure S5) by overlaying the common nuclei (excluding H atoms, the CH_3_ in **5**, and Br in **11**), we found that the root-mean-square deviation in atomic positions between **5** and **11** was 0.0224 Å and between **5** and **13** was 0.0373 Å, with maximum deviations of 0.044 and 0.070 Å for the position of C21 (terminal methyl of the acetonide residue) (see atomic numbering for **5**, **11**, and **13** in Figure 1a, Figure 2a, and Figure 3a). Consistent with this, the dihedral angles between the cyclohexene and dioxolane rings were 76.78(11)°, 77.4(4)°, and 76.71(16)° in **5**, **11**, and **13**, respectively. This implied that the substitution of CH_3_ for H or Br did not have an effect on the molecular conformation. Moreover, the three compounds showed the same strong intermolecular H-bond interactions connecting O4‒H4···3^i^ and O5‒H5···O4^i^ as well as a weaker C7A‒H7A···O5^ii^ interaction, as shown in Table S3 for the three compounds (symmetry codes: (i) −x, y + 1/2, −z + 3/2; (ii) x + 1, y, z). These interactions defined double-layer planes of molecules parallel to the a–b plane, as shown in Figure S6. Considering the molecular shape and interactions, compounds **5** and **13** were clearly isostructural, with a change in the c-axis length that reflected the change in the size of the methyl group with respect to the H atom in C7. Indeed, the substituents at C7 (CH_3_, Br, and H) pointed outwards from the double planes; therefore they had a decisive influence on the interplanar distance and interplanar interactions among parallel planes, consequently explaining the expansion of the c-axis observed in the CH_3_- and Br-substituted molecules. Both weakly interacting CH_3_ and H substituents allowed for a symmetric arrangement of molecules, making the crystals orthorhombic. In compound **11**, for which Br replaced CH_3_, an expanded c-axis was also observed when two unit cells were considered (2 × c = 23.5028 Å). Br atoms in parallel planes showed a different set of interactions to H or CH_3_ that shifted the relative positions of the planes and broke two of the 2_1_ symmetry axes present in **5** and **13**, making the monoclinic structure P2_1_ instead of orthorhombic P2_1_2_1_2_1_, keeping the β angle very close to 90°. These interactions for the bromo-conduritol molecules were of dipolar nature, as Br became close to O1 of a symmetry-related molecule (Br···O1^i^ distance of 3.329 Å related by symmetry operation (i): −x, 1/2 + y, −z), while the methyl group and H atom in **5** and **13** were more than 3.5 Å away from O1. No halogen bonds seemed to be present, as the shortest Br–Br distance was 4.43 Å. This suggests that, although the space-group symmetries differed, the three compounds were isostructural.

The Oak Ridge Thermal Ellipsoid Plot (ORTEP) plots of conduritol C (**14**) and its methyl (**6**) and bromo (**12**) derivatives at C5 are shown in Figure 1b, Figure 2b and Figure 3b, respectively (for a direct visual comparison of the three molecules, see Figure S7). The three compounds crystallized in the hexagonal P6_1_ space group and were isostructural. Unit-cell parameters and additional crystallographic information for the three compounds can be found in Tables S4, S5, and S6. The three molecules showed identical conformations, with root-mean-square deviations of the atomic positions of methyl- and bromo-conduritol common nuclei with conduritol C of 0.0319 and 0.0335 Å and with maximum deviations of 0.070 and 0.057 Å for C5, respectively. The conformationally flexible cyclohexene ring adopted a half-chair conformation with the R substituent; O1, O2, and O4 in equatorial positions; and only O3 in the axial position. Packing of the molecules was directed by strong H-bonds among hydroxyl groups. O1‒H1···O4^i^, O2‒H2···O2^ii^, O3‒H3···O4^iii^, and O4‒H4···O1^iv^ connected symmetry-related molecules (symmetry codes: (i) x − y, x, −5/6 + z; (ii) 1 − y, 1 + x − y, 1/3 + z; (iii) 1 − x, 1 − y, −1/2 + z; (iv) y, −x + y, −1/6 + z; (v) x, y, 1 + z). The same H-bond pattern was observed in the three compounds, for which a three-dimensional network of interactions was found. Table S7 contains the main interactions common to the three molecules. The hexagonal crystal structure determined a very interesting packing arrangement of molecules, leaving voids of sizes related to the size of R, but this discussion is beyond the scope of this report and will be published in due course. 

## 3. Materials and Methods

All commercially available reagents were purchased and used without further purification (Sigma-Aldrich, St. Louis, MO, USA). All solvents were purified by distillation and dried under classical conditions. All reactions were monitored by thin-layer chromatography (TLC) performed on a 0.25 mm silica gel precoated sheet (POLYGRAM SIL G/UV) by Macherey-Nagel (Duren, Germany) using Ultra Violet (UV) light or spray with anisaldehyde solution as visualizing agents. Flash column chromatography was carried out with silica gel (spherical, neutral, 63–210 µm grade). Yields refer to chromatographically and spectroscopically homogeneous materials. Infrared spectra were recorded on neat samples (KBr disks) using a Shimadzu FT/IR-8101 (Kyoto, Japan). Melting points were measured using a melting-point apparatus (Gallenkamp, London, UK) and are uncorrected. Optical rotations were measured in a Kruss Optronic P8000 polarimeter (Berlin, Germany) at Polo Tecnológico de Pando (Facultad de Química) using a 0.5 dm cell; [α]_D_ values are given in units of deg·cm^2^·g^−1^, and concentration values are expressed in grams per 100 mL. ^1^H-NMR spectra (400 MHz) and ^13^C-NMR (100 MHz) were recorded in the indicated solvent on a Bruker Advance DPX 400 MHz spectrometer (Mundelein, IL, USA). Chemical shifts are reported in delta (δ) units, parts per million (ppm). Chemical shifts for ^1^H-NMR spectra are given relative to signals for internal tetramethylsilane (0 ppm). Chemical shifts for ^13^C-NMR spectra are given relative to the signal for CD_3_OD (49.0 ppm). Multiplicities are reported by the following abbreviations: s (singlet), d (doublet), t (triplet), m (multiplet), and dd (double doublet). Coupling constants (*J*) are represented in hertz (Hz). X-ray diffraction data was obtained using a Bruker D8 Venture diffractometer (Mundelein, IL, USA) with a PHOTON100 CMOS detector and with an INOCOATEC Microfocus CuKα (Berkshire, UK) radiation source (λ = 1.54178 Å) at room temperature.

Compound **13** was obtained according to [19], and spectroscopic data matched to those previously reported. 

### 3.1. General Procedure for the Synthesis of Diols ***5*** and ***11***

The corresponding iodohydrin (1.12 mmol) was dissolved in THF (20 mL), and a solution of KOH (4.48 mmol) in water (8 mL) was added at 0 °C. The crude reaction mixture was stirred at room temperature, and it was then brought to reflux for 12 h (monitored by TLC). The solution was extracted with dichloromethane (3 × 25 mL). The organic layer was washed with water (25 mL) and then with 10% HCl solution to pH 7. Finally, it was washed with brine, dried over sodium sulfate, and concentrated under vacuum to afford a yellow oil, which was purified by flash column chromatography using 7:3 ethyl acetate/hexane to afford diols **5** (56%) and **11** (72%) as white crystalline solids. Crystals suitable for X-ray structure analysis were obtained by dissolving the sample in the minimum volume of ethyl acetate, adding hexanes until the solution became slightly turbid, and slowly evaporating the solvent at room temperature. 

Spectroscopic data of compounds **5** [20] and **11** [15] matched to those previously reported.

### 3.2. General Procedure for the Synthesis of Conduritol Derivatives 

The corresponding acetonide (0.21 mmol) was dissolved in methanol (1 mL), and Dowex 50 resin (200 mg) was added to the solution. The reaction mixture was stirred overnight at r.t. The resin was filtered off, and the filtrate was concentrated to afford the corresponding deprotected conduritol. The crude residue was purified by flash column chromatography using ethyl acetate with 10% methanol. Crystals suitable for X-ray structure analysis were obtained by dissolving the sample in the minimum volume of ethyl acetate with 10% methanol, adding hexanes until the solution became slightly turbid, and slowly evaporating the solvent at room temperature. 

*(−)-Methyl-conduritol C* (**6**) (83%), white solid: [α]${}_{D}^{20}$ = −45 (*c* 0.02, MeOH); Infrared = 3320, 2910, 2850, 1030 cm^−1^; ^1^H-NMR (400 MHz, CD_3_OD) δ 1.78 (s, 3H), 3.54 (dd, *J* = 6.8, 2.2 Hz, 1H), 3.97–4.04 (m, 2H), 4.21 (m, 1H), 5.39 (m, 1H); ^13^C-NMR (100 MHz, CD_3_OD) δ 19.6, 71.1, 71.9, 73.2, 76.2, 125.7, 137.5; Melting Point = 143.1–144.0 °C.

*(−)-Bromo-conduritol C* (**12**) (90%), white solid: [α]${}_{D}^{20}$ = −50 (*c* 0.01, MeOH); Infrared = 3340, 1660, 1395, 1030 cm^−1^; ^1^H-NMR (400 MHz, CD_3_OD) δ 3.63 (dd, *J* = 6.8, 2.1 Hz, 1H), 4.09 (dd, *J* = 3.9, 2.1 Hz, 1H), 4.21 (m, 1H), 4.26 (dt, *J* = 6.8, 2.7, 2, 7 Hz, 1H), 6.10 (dd, *J* = 2.8, 1.5 Hz, 1H); ^13^C-NMR (100 MHz, CD_3_OD) δ 71.5, 72.8, 73.1, 75.4, 127.7, 133.0; Melting Point = 129–131 °C. 

*(−)-Conduritol C* (**14**) (92%), white solid: [α]${}_{D}^{20}$ = −205 (*c* 0.2, MeOH); MP = 127–129 °C. Spectroscopic data matched to those previously reported [8].

### 3.3. X-ray Crystallographic Analysis

Single crystals of **6**, **12**, and **14** were obtained as hexagonal or rectangular parallelepipeds on the walls of the rota-evaporation flask during the last purification step, while those of **11** and **13** were grown from a hexane/ethylacetate solution. Crystal information, data collection, and structure determination results are listed in the supplementary information. Cambridge Crystallographic Data Centre (CCDC) numbers 1846151–1846155 contain the supplementary crystallographic data for **6**, **11**, **12**, **13**, and **14**, respectively. The data can be obtained free of charge from the CCDC via www.ccdc.cam.ac.uk/structures.

## 4. Conclusions

In summary, we developed a general method for the synthesis of (−)-conduritol C derivatives starting from commercially available monosubstituted benzenes. This simple and efficient short synthesis (four steps, with 46% for bromo-conduritol C) ensures the desired stereochemical configuration of the conduritol C structure by means of an enzymatic transformation and enantioselective ring opening of epoxide. 

Additionally, our method can be generalized to a variety of conduritol C derivatives, starting from the numerous *cis*-1,2-cyclohexadienediol derivatives previously reported, which are obtained by biotransformation [21].

The three conduritol derivatives and acetonide precursors show isostructurality, as a result of a particular crystal packing that conserves the most significant intermolecular interactions unaffected by the change of the R group bonded to C5 (C7 in the acetonides). 

## Supplementary Materials

The following are available online: Figure S1: ^1^H-NMR methyl-conduritol (**6**); Figure S2: ^13^C-NMR methyl-conduritol (**6**); Figure S3: ^1^H-NMR bromo-conduritol (**12**); Figure S4: ^13^C-NMR bromo-conduritol (**12**); Figure S5: ORTEP X-ray structures corresponding to compounds **13** (a), **5** (b), and **11** (c); Figure S6: The crystal packing of compounds **5** (a), **11** (b), and **13** (c), viewed along c-axis direction showing the strong O‒H·∙·O hydrogen bond interactions along [010]; Figure S7: ORTEP X-ray structures of compounds **14** (a), **6** (b), and **12** (c); Table S1: Sample, crystal data, data collection, and refinement for compound **11**; Table S2: Sample, crystal data, data collection, and refinement for compound **13**; Table S3: Hydrogen bonds for compounds **5** (extracted from [12]), **11**, and **13**; Table S4: Sample, crystal data, data collection, and refinement for compound **6**; Table S5: Sample, crystal data, data collection, and refinement for compound **12**; Table S6: Sample, crystal data, data collection, and refinement for compound **14**; Table S7: Hydrogen bonds for compounds **6**, **12**, and **14**.

## Abbreviations

DMP	dimetoxypropane
NIS	*N*-Iodosuccinimide
DME	Dimetoxyethane
THF	Tetrahydrofurane
