# Supplementary material for: General Method for the Synthesis of (−)-Conduritol C and Analogs from Chiral Cyclohexadienediol Scaffolds
Source: Molecules. 2018 Jul 6;23(7):1653. doi: 10.3390/molecules23071653 (PMC6100410; doi:10.3390/molecules23071653)
Supplement: Supplementary file 1 [file molecules-23-01653-s001.pdf]

SUPPORTING INFORMATION

Article

General Method for the Synthesis of (−)-Conduritol C and Analogs from Chiral Cyclohexadienediol Scaffolds

Gaurao D. Tibhe [1], Mario A. Macías [2,3], Valeria Schapiro [1], Leopoldo Suescun [2] and Enrique Pandolfi [1,*]

[1] Laboratorio de Síntesis Orgánica, Departamento de Química Orgánica, Facultad de Química, Av. Gral. Flores 2124, Universidad de la República, Montevideo, CP11800, Uruguay; gaurao.tibhe@gmail.com (G.D.T.); vschapir@fq.edu.uy (V.S.)

[2] Cryssmat-Lab/DETEMA, Facultad de Química, Av. Gral. Flores 2124, Universidad de la República, Montevideo, CP11800, Uruguay; ma.maciasl@uniandes.edu.co (M.A.M.); Leopoldo@fq.edu.uy (L.S.)

[3] Department of Chemistry, Universidad de los Andes, Carrera 1 N8 18 A-12, Bogotá 111711, Colombia

* Correspondence: epandolf@fq.edu.uy; Tel.: +598-2-9247881

Academic Editor: Laura Palombi and Antonio Massa

Received: 31 May 2018; Accepted: 1 July 2018; Published: date

NMR Spectroscopic data

(-)-Methyl-conduritol C (**6**) :

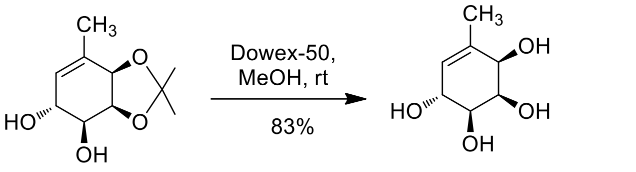

methyl conduritol C (6)

Figure S1. NMR ^{1}H methyl –conduritol C (**6**)

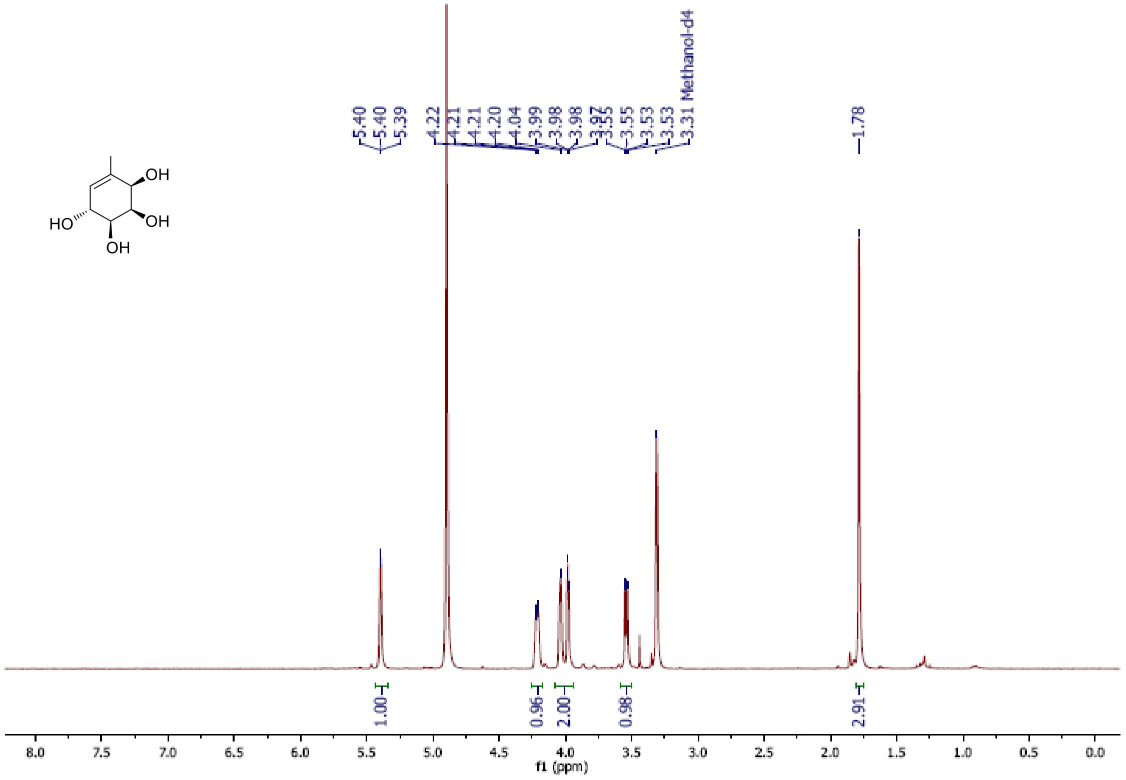

Figure S2. NMR ^{13}C methyl –conduritol C(**6**)

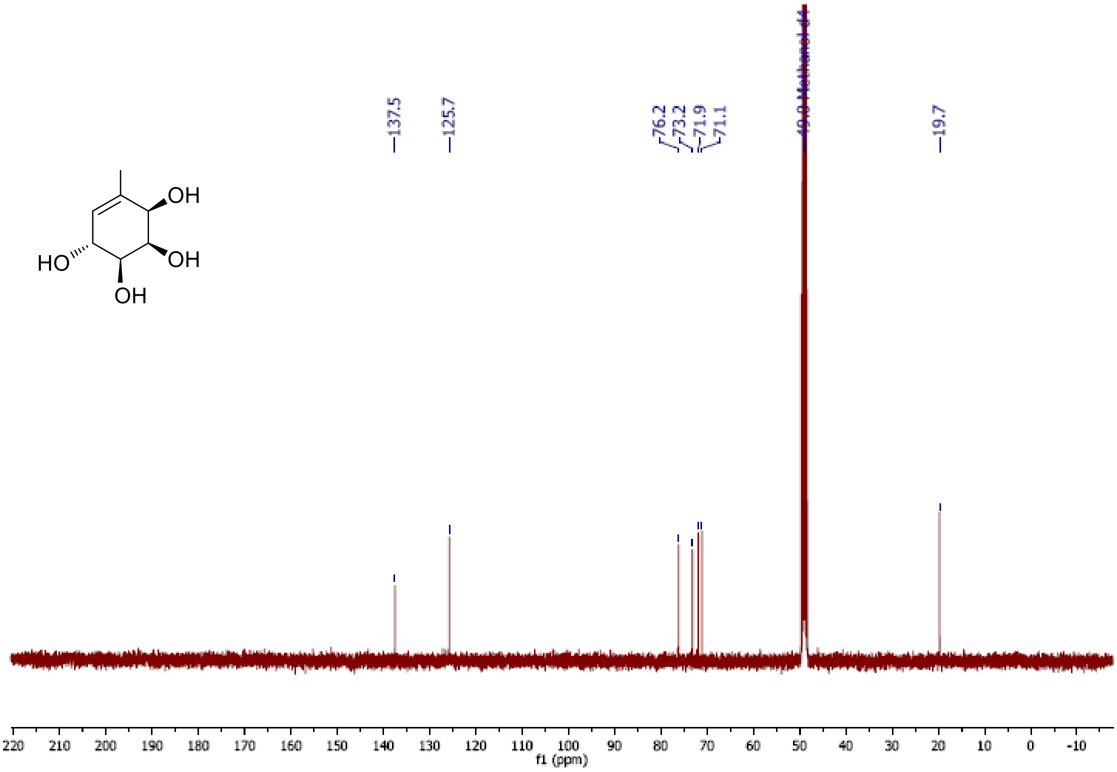

(-)-Bromo-conduritol C (**12**)

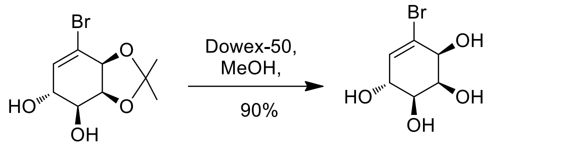

bromo-conduritol C (12)

Figure S3. NMR ^{1}H bromo –conduritol (**12**)

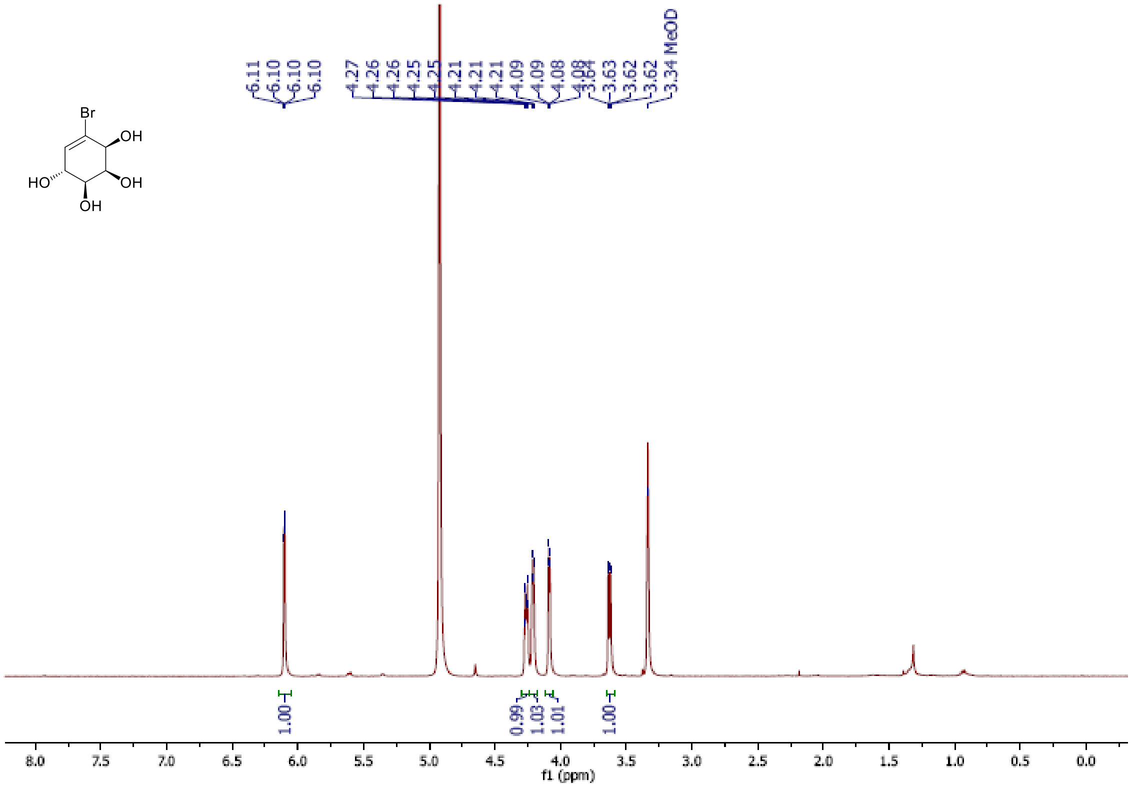

Figure S4. NMR ^{13}C bromo –conduritol C (**12**)

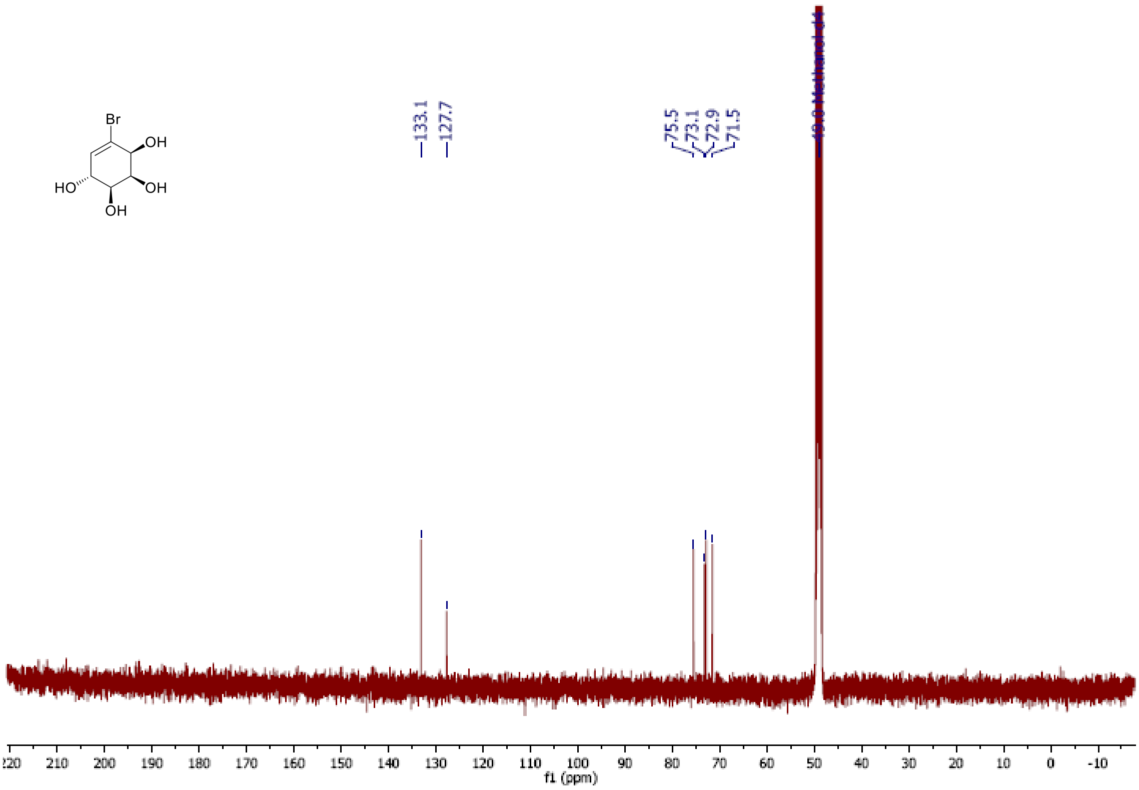

Table S1. Sample, crystal data, data collection and refinement for compound **11**

**Chemical formula**	$C_9H_{13}BrO_4$	
**Formula weight**	264.10 g/mol	
**Temperature**	296(2) K	
**Wavelength**	1.54178 Å	
**Crystal size**	0.17 x 0.11 x 0.05 mm	
**Crystal system**	monoclinic	
**Space group**	$P2_1$	
**Unit cell dimensions**	a = 6.1570(2) Å	α = 90°
	b = 7.4749(3) Å	β = 93.267(3)°
	c = 11.7514(4)Å	γ = 90°
**Volume**	539.96(3) $Å^3$	
**Z**	2	
**Density (calculated)**	1.631 g/cm^3	
**Absorption coefficient**	5.12 mm^{-1}	
**F(000)**	268	

**Theta range for data collection**	3.77 to 74.73°
**Index ranges**	$-7\leq h\leq 7$, $-9\leq k\leq 8$, $-14\leq l\leq 14$
**Reflections collected**	12499
**Independent reflections**	2193 [R(int) = 0.0705]
**Coverage of independent reflections**	99.7%
**Absorption correction**	multi-scan
**Max. and min. transmission**	0.7870 and 0.4830
**Refinement method**	Full-matrix least-squares on F^2
**Refinement program**	SHELXL-2014/6 (Sheldrick, 2014)
**Function minimized**	Σ w(F_o^2 - F_c^2)^2
**Data / restraints / parameters**	2193 / 1 / 132
**Goodness-of-fit on F^2**	1.064
**Final R indices**	1757 data; $I>2\sigma(I)$ R1 = 0.0483,wR2 = 0.1400
	all data R1 = 0.0644, wR2 = 0.1540
**Weighting scheme**	$w=1/[\sigma^2(F_o^2)+0.1000P^2]$<br>where $P=(F_o^2+2F_c^2)/3$
**Absolute structure parameter**	-0.05(3)
**Largest diff. peak and hole**	0.470 and -0.488 $eÅ^{-3}$
**R.M.S. deviation from mean**	0.067 $eÅ^{-3}$

Table S2. Sample, crystal data, data collection and refinement for compound **13**

**Chemical formula**	$C_9H_{14}O_4$	
**Formula weight**	186.20 g/mol	
**Temperature**	296(2) K	
**Wavelength**	1.54178 Å	
**Crystal size**	0.036 x 0.124 x 0.137 mm	
**Crystal system**	orthorhombic	
**Space group**	$P2_12_12_1$	
**Unit cell dimensions**	a = 6.1358(5) Å	α = 90°
	b = 7.5083(5) Å	β = 90°
	c = 20.5776(14) Å	γ = 90°
**Volume**	948.00(12) $Å^3$	
**Z**	4	
**Density (calculated)**	1.305 g/cm^3	
**Absorption coefficient**	0.858 mm^{-1}	
**F(000)**	400	

**Theta range for data collection**	4.30 to 68.54°	
**Index ranges**	$-7 \le h \le 7$, $-9 \le k \le 8$, $-24 \le l \le 24$	
**Reflections collected**	11040	
**Independent reflections**	1736 [R(int) = 0.0753]	
**Coverage of independent reflections**	99.4%	
**Absorption correction**	multi-scan	
**Max. and min. transmission**	0.970 and 0.891	
**Refinement method**	Full-matrix least-squares on F^2	
**Refinement program**	SHELXL-2016/6 (Sheldrick, 2016)	
**Function minimized**	Σ w(F_o^2 - F_c^2)^2	
**Data / restraints / parameters**	1736 / 0 / 126	
**Goodness-of-fit on F^2**	1.066	
**Final R indices**	1491 data; I>2σ(I)	R1 = 0.0381,wR2 = 0.0770
	all data	R1 = 0.0529, wR2 = 0.0807
**Weighting scheme**	w=1/[σ^2(F_o^2)+0.2457P]<br>where P=(F_o^2+2F_c^2)/3	
**Absolute structure parameter**	0.0(2)	
**Largest diff. peak and hole**	0.112 and -0.136 e$Å^{-3}$	
**R.M.S. deviation from mean**	0.031 e$Å^{-3}$	

Figure S5. ORTEP X-ray structures correponding to compounds **13(a)**, **5(b),** and **11(c)**.

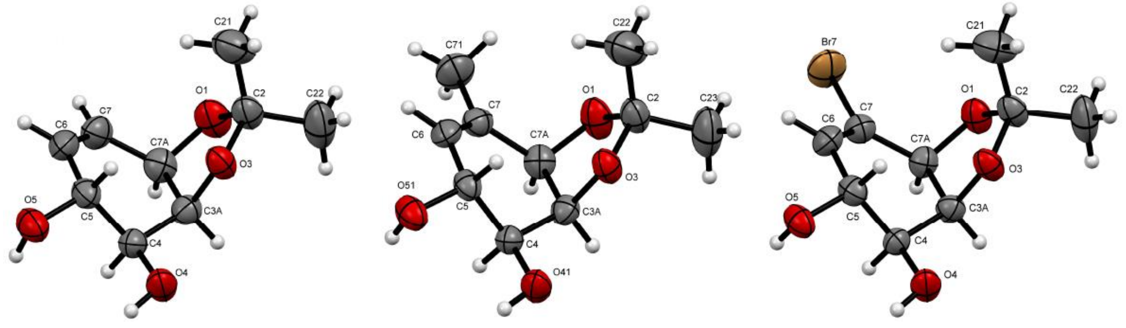

a b c

Table S3. Hydrogen bonds for compounds 5 (extracted from [12]), **11** and **13**

**D–H···*A***	**D–*A***	**H···*A***	**D···*A***	**D–H···*A***
**Compound 5**				
O41–H41···O3^{i}	0.82(3)	2.04(3)	2.850(2)	172(3)
O51–H51···O41^{i}	0.80(3)	2.04(3)	2.826(2)	167(2)
C7A–H7A···O51^{ii}	0.98	2.44	3.299(3)	146
Symmetry codes: (i) –*x*, *y*+1/2, -*z*+3/2; (ii) *x*+1, *y*, *z*.				
**Compound 11**				
O4–H4···O3^{i}	0.82	2.01	2.826(7)	174
O5–H5···O4^{i}	0.82	2.06	2.822(7)	155
C7A–H7A···O5^{ii}	0.98	2.39	3.262(8)	148
Symmetry codes: (i) 1-*x*, 1/2+*y*, 1-*z*; (ii) -1+*x*, *y*, *z*				
**Compound 13**				
O4–H4···O3^{i}	0.88(3)	1.97(3)	2.847(3)	172(3)
O5–H5···O4^{i}	0.88(3)	1.97(3)	2.835(3)	170(4)
C7A–H7A···O5^{ii}	0.98	2.52	3.353(4)	143
Symmetry codes: (i) 1-*x*,-1/2+*y*,1/2-*z*; (ii) -1+*x*, *y*, *z*.				

Table S4. Sample, crystal data, data collection and refinement for compound **6**

**Chemical formula**	$C_7H_{12}O_4$	
**Formula weight**	160.17 g/mol	
**Temperature**	296(2) K	
**Wavelength**	1.54178 Å	
**Crystal size**	0.036 x 0.098 x 0.186 mm	
**Crystal system**	hexagonal	
**Space group**	$P6_1$	
**Unit cell dimensions**	a = 16.5762(3) Å	α = 90°
	b = 16.5762(3) Å	β = 90°
	c = 4.94850(10) Å	γ = 120°
**Volume**	1177.54(5) $Å^3$	
**Z**	6	
**Density (calculated)**	1.354 g/cm^3	
**Absorption coefficient**	1.355 mm^{-1}	
**F(000)**	516	

**Theta range for data collection**	3.08 to 80.33°	
**Index ranges**	$-21 \leq h \leq 19$, $-20 \leq k \leq 21$, $-6 \leq l \leq 6$	
**Reflections collected**	20213	
**Independent reflections**	1702 [R(int) = 0.1371]	
**Coverage of independent reflections**	99.1%	
**Absorption correction**	multi-scan	
**Max. and min. transmission**	0.967 and 0.844	
**Refinement method**	Full-matrix least-squares on F^2	
**Refinement program**	SHELXL-2016/6 (Sheldrick, 2016)	
**Function minimized**	Σ w(F_o^2 - F_c^2)^2	
**Data / restraints / parameters**	1702 / 1 / 110	
**Goodness-of-fit on F^2**	1.046	
**Final R indices**	1424 data; I>2σ(I)	R1 = 0.0562,wR2 = 0.1330
	all data	R1 = 0.0694, wR2 = 0.1423
**Weighting scheme**	w=1/[σ^2(F_o^2)+(0.0903P)^2]<br>where P=(F_o^2+2F_c^2)/3	
**Absolute structure parameter**	0.2(5)	
**Largest diff. peak and hole**	0.199 and -0.212 e$Å^{-3}$	
**R.M.S. deviation from mean**	0.051 e$Å^{-3}$	

Table S5. Sample, crystal data, data collection and refinement for compound **12**

**Chemical formula**	$C_6H_9BrO_4$	
**Formula weight**	225.04 g/mol	
**Temperature**	296(2) K	
**Wavelength**	1.54178 Å	
**Crystal size**	0.016 x 0.033 x 0.152 mm	
**Crystal system**	hexagonal	
**Space group**	$P6_1$	
**Unit cell dimensions**	a = 16.7667(5) Å	α = 90°
	b = 16.7667(5) Å	β = 90°
	c = 4.9262(2) Å	γ = 120°
**Volume**	1199.33(9) $Å^3$	
**Z**	6	
**Density (calculated)**	1.869 g/cm^3	
**Absorption coefficient**	6.784 mm^{-1}	
**F(000)**	672	

**Theta range for data collection**	3.04 to 78.95°	
**Index ranges**	$-21\leq h\leq 21$, $-20\leq k\leq 21$, $-5\leq l\leq 6$	
**Reflections collected**	29055	
**Independent reflections**	1682 [R(int) = 0.0865]	
**Coverage of independent reflections**	98.9%	
**Absorption correction**	multi-scan	
**Max. and min. transmission**	0.844 and 0.967	
**Refinement method**	Full-matrix least-squares on F^2	
**Refinement program**	SHELXL-2014/6 (Sheldrick, 2014)	
**Function minimized**	Σ w(F_o^2 - F_c^2)^2	
**Data / restraints / parameters**	1682 / 1 / 104	
**Goodness-of-fit on F^2**	1.171	
**Final R indices**	1559 data; I>2σ(I)	R1 = 0.0699,wR2 = 0.1402
	all data	R1 = 0.0775, wR2 = 0.1431
**Weighting scheme**	w=1/[σ^2(F_o^2)+(0.0269P)^2+11.9938P]<br>where P=(F_o^2+2F_c^2)/3	
**Absolute structure parameter**	-0.03(9)	
**Largest diff. peak and hole**	0.549 and -0.621 e$Å^{-3}$	
**R.M.S. deviation from mean**	0.096 e$Å^{-3}$	

Table S6. Sample, crystal data, data collection and refinement for compound **14.**

**Chemical formula**	$C_6H_{10}O_4$	
**Formula weight**	146.14 g/mol	
**Temperature**	296(2) K	
**Wavelength**	1.54178 Å	
**Crystal size**	0.036 x 0.068 x 0.152 mm	
**Crystal system**	hexagonal	
**Space group**	$P6_1$	
**Unit cell dimensions**	a = 16.454(10) Å	α = 90°
	b = 16.454(10) Å	β = 90°
	c = 4.925(3) Å	γ = 120°
**Volume**	1154.7(16) $Å^3$	
**Z**	6	
**Density (calculated)**	1.261 g/cm^3	
**Absorption coefficient**	0.916 mm^{-1}	
**F(000)**	468	

**Theta range for data collection**	3.10 to 79.08°	
**Index ranges**	$-20\leq h\leq 20$, $-20\leq k\leq 16$, $-6\leq l\leq 6$	
**Reflections collected**	12398	
**Independent reflections**	1608 [R(int) = 0.0629]	
**Coverage of independent reflections**	98.4%	
**Absorption correction**	multi-scan	
**Max. and min. transmission**	0.968 and 0.873	
**Refinement method**	Full-matrix least-squares on F^2	
**Refinement program**	SHELXL-2016/6 (Sheldrick, 2016)	
**Function minimized**	Σ w(F_o^2 - F_c^2)^2	
**Data / restraints / parameters**	1608 / 1 / 96	
**Goodness-of-fit on F^2**	1.310	
**Final R indices**	1578 data; I>2σ(I)	R1 = 0.1195,wR2 = 0.3201
	all data	R1 = 0.1260, wR2 = 0.3342
**Weighting scheme**	w=1/[σ^2(F_o^2)+(0.0951P)^2+5.1617P]<br>where P=(F_o^2+2F_c^2)/3	
**Absolute structure parameter**	0.4(6)	
**Largest diff. peak and hole**	0.283 and -0.278 e$Å^{-3}$	
**R.M.S. deviation from mean**	0.072 e$Å^{-3}$	

Figure S6. The crystal packing of compounds **5** (a), **11**(b) and c) **13**(c), viewed along c axis direction showing the strong O–H···O hydrogen-bond interactions along [010].

(a)

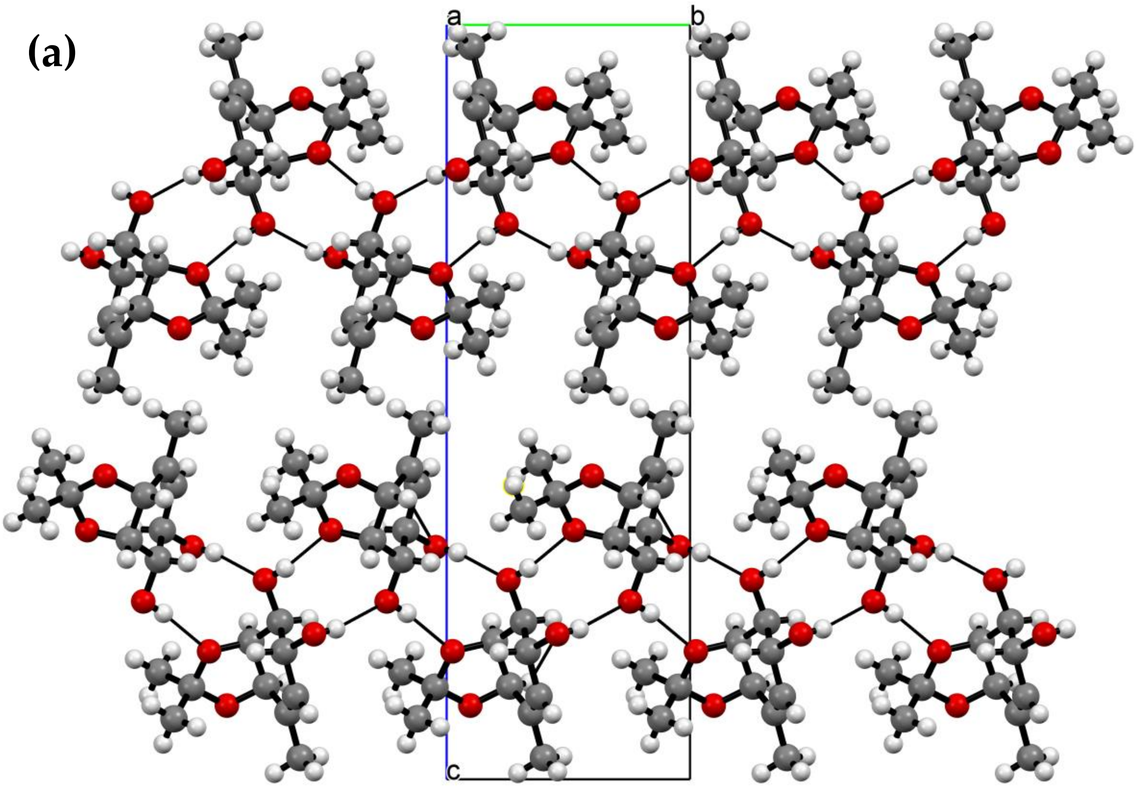

(b)

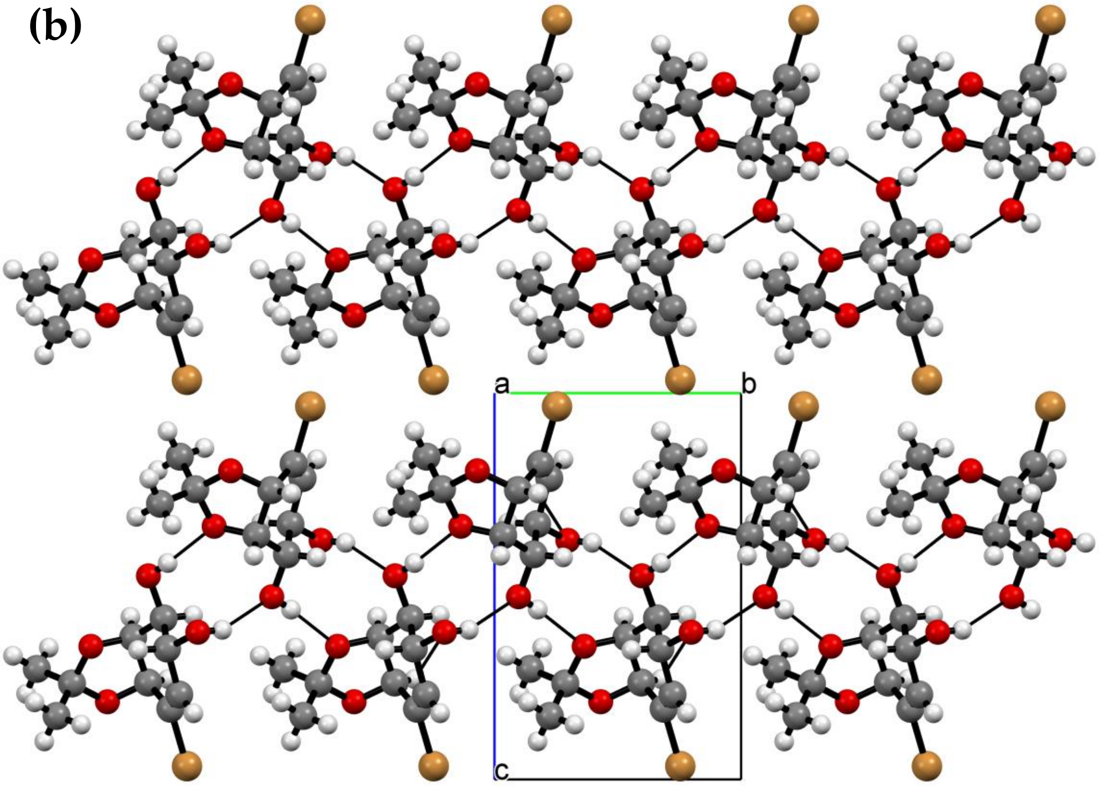

(c)

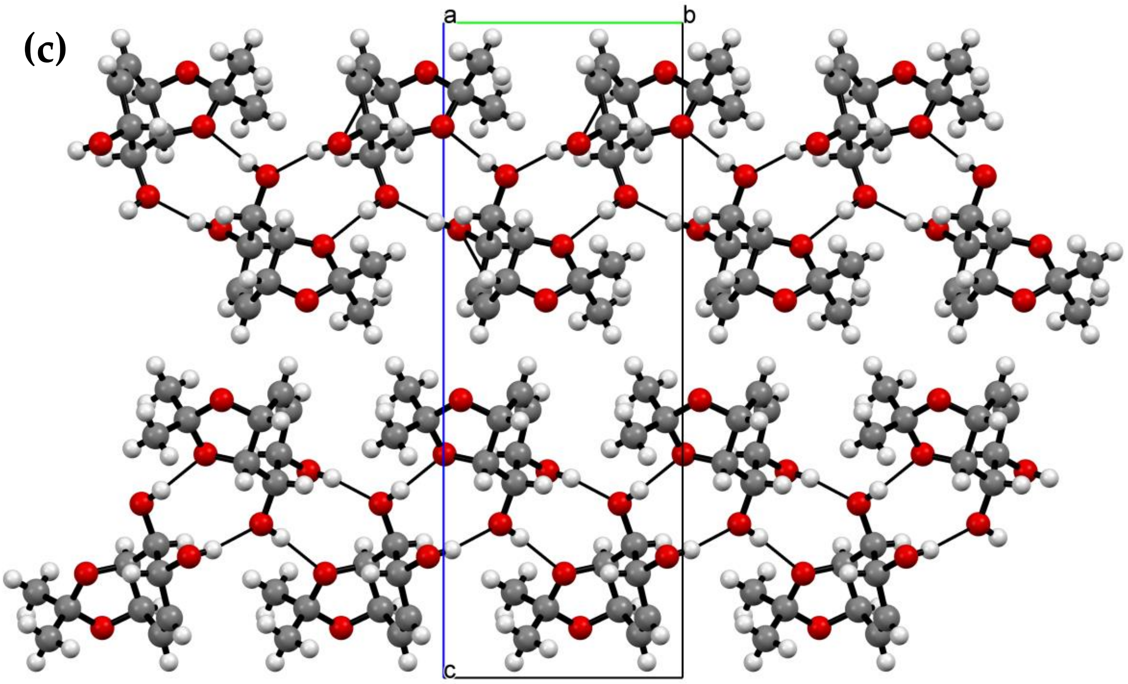

Figure S7. ORTEP X-ray structures of compounds **14** (a), **6**(b), and **12** (c).

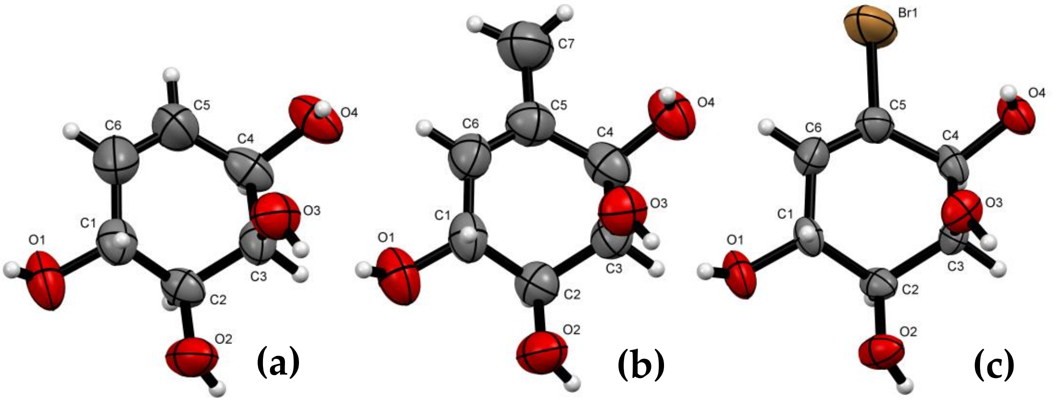

Table S7. Hydrogen bonds for compounds **6**, **12** and **14**

**D–H···*A***	**D–*A***	**H···*A***	**D···*A***	**D–H···*A***
**Compound 6 (metil-conduritol C)**				
O1–H1···O4[i]	0.83(4)	2.01(3)	2.826(4)	172(4)
O2–H2···O2[ii]	0.83(3)	1.93(3)	2.722(5)	160(4)
C3–H3A···O1[ii]	0.98	2.57	3.448(7)	150
O3–H3···O4[iii]	0.82(4)	2.06(4)	2.871(5)	169(3)
O4–H4···O1[iv]	0.83(2)	1.99(2)	2.812(4)	169.7(16)
C4–H4A···O3[v]	0.98	2.40	3.232(5)	143
**Compound 12 (bromo-conduritol C)**				
O1–H1···O4[i]	0.82	2.05	2.864(13)	176
O2–H2···O2[ii]	0.82	1.93	2.738(11)	170
C3–H3A···O1[ii]	0.98	2.57	3.462(14)	151
O3–H3···O4[iii]	0.82	2.04	2.836(11)	165
O4–H4···O1[iv]	0.82	2.02	2.831(12)	169
C4–H4A···O3[v]	0.98	2.36	3.190(12)	143
**Compound 14 (conduritol C)**				
O1–H1···O4[i]	0.82	1.97	2.773(10)	167
O2–H2···O2[ii]	0.82	1.92	2.713(9)	163
O3–H3···O4[iii]	0.82	2.15	2.935(9)	161
O4–H4···O1[iv]	0.82	1.95	2.731(10)	158' 91' 355
C4–H4A···O3[v]	0.98	2.51	3.290(10)	136

Symmetry codes: (i) *x*-*y*,*x*,-5/6+*z*; (ii) 1-*y*,1+*x*-*y*,1/3+*z*; (iii) 1-*x*,1-*y*,-1/2+*z*; (iv) *y*,-*x*+*y*,-1/6+*z*; (v) *x*,*y*,1+*z*
